# Revealing the kinetics of *Leishmania chagasi* infection in the male genital system of hamsters

**DOI:** 10.1186/s40249-016-0122-0

**Published:** 2016-03-29

**Authors:** Amanda P. N. Quintal, Bruna C. Borges, Paula C. Brígido, Rebecca T. Silva, Ana F. Notário, Marlus A. Santos, Maria A. de Souza, Fernanda G. O. Nascimento, Antônio V. Mundim, Guilherme M. J. Costa, André B. Vasconcelos, Claudio V. da Silva

**Affiliations:** Instituto de Ciências Biomédicas, Universidade Federal de Uberlândia, Uberlândia, Minas Gerais Brasil; Hospital Veterinário, Universidade Federal de Uberlândia, Uberlândia, Minas Gerais Brasil; Departamento de Ciências Biomédicas, Universidade Federal de Minas Gerais, Belo Horizonte, Brasil; Universidade de Uberaba, Uberaba, Minas Gerais Brasil; Departamento de Imunologia – Instituto de Ciências Biomédicas, Universidade Federal de Uberlândia, Campus Umuarama, Uberlândia, MG Brasil

**Keywords:** *Leishmania chagasi*, Extracellular amastigotes, Testis, Epididymis, Immune response, Testosterone

## Abstract

**Background:**

Leishmaniasis causes alterations and lesions in the genital system, which leads to azoospermia and testicular atrophy in animals during the chronic phase of the infection. The aim of this study was to reveal the kinetics of *Leishmania chagasi* infection in the genital system of male golden hamsters (*Mesocricetus auratus*).

**Methods:**

Animals were intraperitoneally inoculated with amastigotes from *L. chagasi*. At different time points animals were euthanized and genital organs processed for histo-pathological, qPCR, cytokines and testosterone detection assays.

**Results:**

Our results showed a high parasite load in testis, followed by an increase of pro-inflammatory cytokines IL1-β, TNF-α and IFN-γ, and testosterone. Subsequently, IL-4 expression was upregulated and basal parasite persistence in testis was observed using the experimental approach.

**Conclusion:**

Extracellular amastigotes migrated to the epididymis posing as a potential major factor of parasite persistence and venereal transmission of *L. chagasi* infection in hamsters.

**Electronic supplementary material:**

The online version of this article (doi:10.1186/s40249-016-0122-0) contains supplementary material, which is available to authorized users.

## Multilingual abstracts

Please see Additional file 1 for the translations of abstract into the six official working languages of the United Nations.

## Findings

*Leishmania chagasi* is an intracellular protozoan that causes visceral leishmaniasis (VL) [1], a potentially fatal human disease that infects the macrophages in the spleen and liver, leading to splenomegaly and hepatomegaly [2]. It is estimated that globally 200,000 to 400,000 new cases and 20,000 to 30,000 deaths occur each year [3].

Visceral leishmaniasis causes alterations and lesions in the genital system [2, 4, 5]. Other authors have previously demonstrated the presence of the *Leishmania* parasite in urine, semen, and reproductive organs of dogs [5–9]. In addition, the possibility of venereal transmission in dogs naturally infected by *L. chagasi* has been proposed [5]. In this study, we aimed to verify the kinetics of *L. chagasi* infection in the genital organs of male golden hamsters.

## Methods

### Animals and parasites

Male golden hamsters (*Mesocricetus auratus*) aged 6 to 8 weeks were kept under standard conditions on a 12-h light, 12-h dark cycle in a temperature-controlled room (25 ± 2 °C), with food and water available *ad libitum*.

*L. chagasi* (MHON/BR/1972/LD) strain was maintained in vivo by inoculation into peritoneal cavity of hamsters. Six weeks post-infection, the hamsters were euthanized and amastigote forms were recovered from their spleens. The spleens were removed, macerated in 4 mL of phosphate-buffered saline (PBS), and centrifuged to remove residual tissue. The purified amastigotes were counted in bright light microscope and used to infect animals for the experimental procedures.

### Experimental infection

Forty-two male golden hamsters were split into six groups of seven hamsters each. Animals were intraperitoneally inoculated with 1 × 10^6^*L. chagasi* amastigotes, and euthanasia was conducted seven, 10, 13, 16, and 19 weeks post-infection. At each of these times, the spleens, livers, testis, and epididymis were removed and split into three equal parts for polymerase chain reaction (PCR), cytokine, testosterone detection, and histological procedures.

### Histological samples

Paraffin-embedded epididymis samples were used in indirect immunofluorescence as follows: First, paraffin was removed with xylene and alcohol, then it was treated with 50 mM of ammonium chloride for one hour, and finally it was blocked with albumin (one egg white qsq in 100 ml of distilled water) for 20 min and with skimmed milk overnight. After this, the samples were incubated with rabbit polyclonal antibody of anti-*L. chagasi* diluted in PGN-saponin (PBS + gelatin + azide) (1: 100) overnight. Finally, the samples were incubated with mouse anti-rabbit IgG Alexa Fluor® 488 (Thermo Fisher Scientific, USA) conjugated antibody (1:200) and TO-PRO®-3 stain (Life Technologies, USA) diluted in PGN-saponin (1:500) for one hour and analyzed using a confocal microscope.

### Conventional and qPCR

Primers 13A (5’ - GGG GTG GAG TCT GGG CGT – 3’) and 13B (5’ - ATT TTA CAC CAA CCC CCA GTT – 3’) were used in conventional and real-time PCR (qPCR) detection. The procedures were performed as described elsewhere [10, 11].

### Cytokines and testosterone detection

Enzyme-linked immunosorbent assay (ELISA) was used to detect the IFN-γ, TNF-α, I-L4, and IL1-β cytokines (BD OpTEIA™, BD Bioscience, San Diego, CA, USA), and testosterone (Testosterone ELISA Kit, Cayman Chemical Company, USA) in the testicle samples. The procedures were performed according to the manufacturers’ instructions.

### Statistical analysis

Statistical analysis was performed using GraphPad Prism 6.0 (GraphPad Software Inc., San Diego, CA, USA). Data were expressed as mean ± standard deviation. An analysis of variance (ANOVA) was performed followed by Bonferroni post-test. *P* <0.05 was considered significant.

### Ethics

Maintenance and care of the animals complied with the guidelines of the laboratory of the Animal Ethics Committee from University of Uberaba. Animal euthanasia was performed in accordance with the American Veterinary Medical Association Guidelines for the Euthanasia of Animals. The research was approved by the Ethics Committee for Animal Experimentation of the University of Uberaba (process CEEA 016004/2014).

## Results and Discussion

**Fig. 1:**
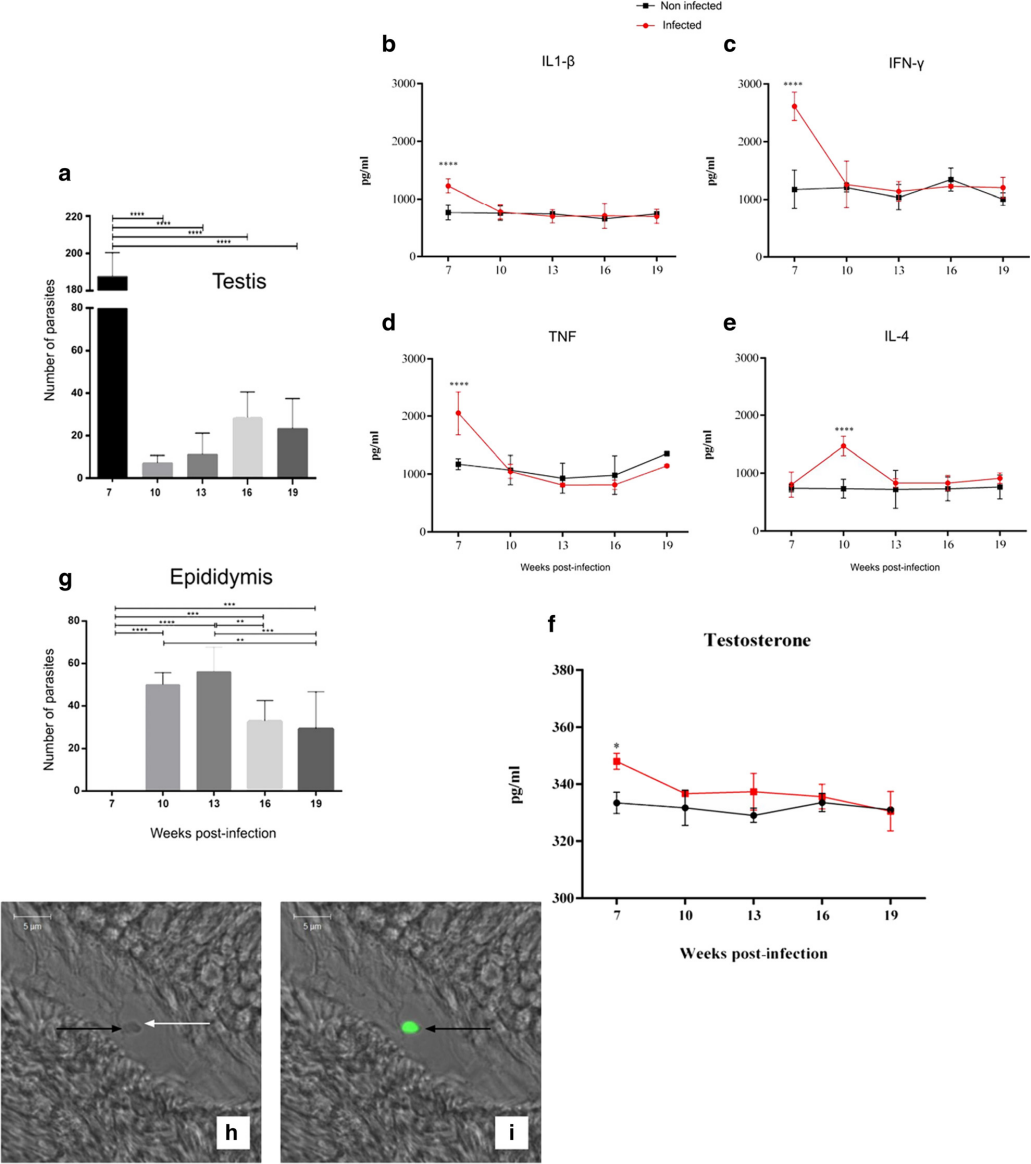
High testis parasitism triggered host immune response and testosterone secretion with parasite migration to epididymis. During week seven post-infection, testis showed a high parasite burden (**a**). Intense parasitism triggered the secretion of pro-inflammatory cytokines (**b**, **c**, and **d**). Secretion of IL-4 (**e**) and progesterone (**f**) controlled immunity and favored parasite persistence. Upon host immune response, extracellular amastigotes migrated to the epididymis (**g**). Representative images of extracellular amastigotes are shown in phase contrast (**h**) and overlay of phase contrast and amastigote staining in green (**i**). Arrows indicate the presence of extracellular amastigotes. ****p < 0.0001; ***p < 0.001; **p < 0.01; *p < 0.05

After confirming animal infection by conventional PCR of DNA extracted from the livers and spleens of the hamsters (data not shown), we evaluated the parasite load in the testis and epididymis. We observed a high parasite load in the testis of infected animals by week seven post-infection (see Fig. 1a). The intensity of tissue parasitism correlated to pro-inflammatory cytokines expression. (see Fig. 1b, c, and d). Interestingly, high levels of pro-inflammatory cytokines were followed by an increased expression of testosterone (see Fig. 1f). Testosterone seems to play an important anti-inflammatory role in the maintenance of testicular immune privilege [12]. The increased testosterone levels observed at 7 week post-infection may have accounted for a basal level of parasite persistence in the testis along the kinetic of infection. Also, testosterone may have induced IL-4 expression at week 10 post-infection (see Fig. 1e).

At week 10 post-infection, we observed a high parasite load in the epididymis, with these levels maintained up to week 13 post-infection (see Fig. 1g). Observing extracellular amastigotes in the infected organs (see Fig. 1h and i).

Authors have demonstrated the presence of *L. chagasi* amastigotes inside testicular macrophages [2]. Additionally, other authors have found intracellular amastigotes in the extraocular striated muscle and in the orbiculari oculi muscle in dogs with patent leishmaniasis [13]. However, this is the first study of extracellular amastigotes in animal tissue. The data suggest that extracellular amastigotes could be major elements involved in infection of genital organs and parasite venereal propagation in animals.

**Fig. 2:**
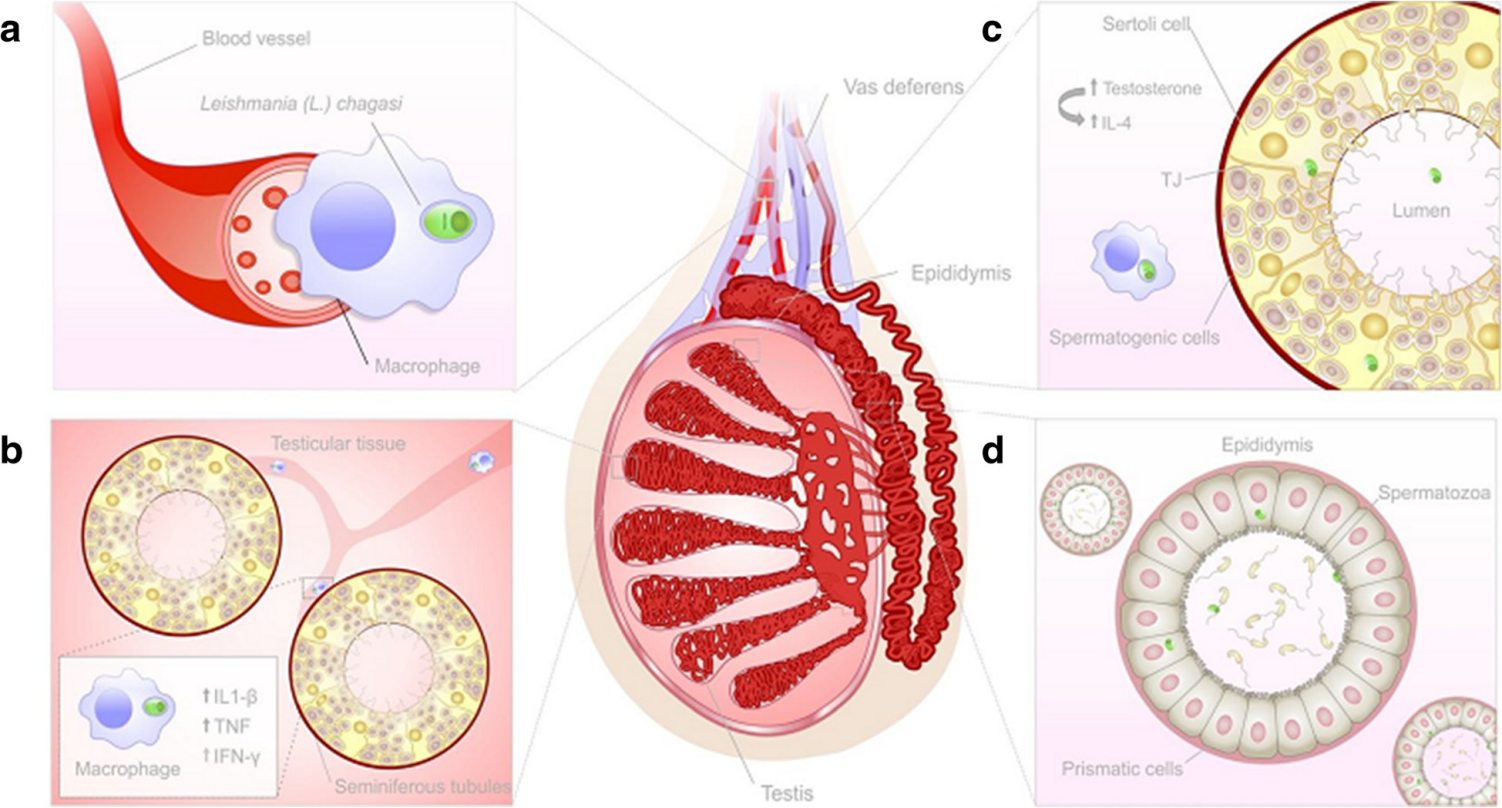
Proposed kinetic of *L. chagasi* infection in the genital system of male hamsters. Parasites reach testis through blood vessels (**a**). Upon the onset of a pro-inflammatory immune response, parasitism is controlled (**b**). The expression of testosterone and IL-4 may have favored the persistence of a low parasite load (**c**). Extracellular amastigotes migrate to the epididymis where they might be adhered to prismatic cells cilia or be released within sperm (**d**)

According to our data, we proposed a kinetic of *L. chagasi* infection of the male genital system. First, parasites may reach the testis through blood vessels. Upon the onset of a pro-inflammatory immune response (T-helper 1 – Th1), parasitism is controlled [14]. However, the expression of testosterone [12] and IL-4 may have favored the persistence of a low parasite load in the testis by establishing a local T-helper 2 (Th2) immune response. In vivo and in vitro studies have established IL-4’s clear role in driving Th2 immunity [15]. This pattern of acquired immunity favors parasite persistence [14]. Meanwhile, extracellular amastigotes migrate to the epididymis, where they might remain adhered to prismatic cells cilia or be released in sperm [5] (see Fig. 2).

## Conclusion

This study revealed the kinetics of *L. chagasi* migration through the male genital system of hamsters. It also provided evidence that extracellular amastigotes may be a major factor of venereal transmission of *Leishmania* in animals.

## Additional file

Additionalfile 1: Multilingual abstracts in the six official working languages of the United Nations. (PDF 356 kb)

## Abbreviations

ELISA: enzyme-linked immunosorbent assay
PBS: phosphate-buffered saline
PCR: polymerase chain reaction
qPCR: real-time PCR
VL: visceral leishmaniasis
