# Supplementary material for: Revealing the kinetics of Leishmania chagasi infection in the male genital system of hamsters
Source: Infect Dis Poverty. 2016 Mar 29;5:29. doi: 10.1186/s40249-016-0122-0 (PMC4812609; doi:10.1186/s40249-016-0122-0)
Supplement: Additionalfile 1: — Multilingual abstracts in the six official working languages of the United Nations. (PDF 356 kb) [file 40249_2016_122_MOESM1_ESM.pdf]

Translation of the abstract into the six official working languages of the United Nations

الكشف عن حركية عدوى الليشمانيا الشاغاسية في الجهاز التناسلي لذكور الهامستر

أماندا ب ن كينتال، برونا س بورخيس، بولا س بريجيدو، ريبيكا ت سيلفا، آنا ف نوتاريو، مارلوس أ سانتوس، ماريا دي سوزا، فرناندا ج و ناسيمنتو، أنطونيو ف مونديم، جويلهيرم م ج كوستا، أندريه ب فاسكونسيلوس، كلاوديو ف سيلفا

ملخص

خلفية: يتسبب داء الليشمانيات في حدوث تعديلات وتمزقات في الجهاز التناسلي، الأمر الذي يؤدي إلى فقد النطاف وضمور الخصية في الحيوانات خلال المرحلة المزمنة من العدوى. وكان الهدف من هذه الدراسة الكشف عن حركية عدوى الليشمانيا الشاغاسية في الجهاز التناسلي لذكور لهامستر الذهبي (*Mesocricetus auratus*).

تم تلقيح الحيوانات داخل الصفاق (الغشاء البريتونى) بليشْمانَات من *L. Chagasi*. وفي نقاط زمنية مختلفة تم قتل الحيوانات بواسطة الموت الرحيم وأُخذت الأعضاء التناسلية لإجراء فحوصات الكشف النسيجية المرضية ، وPCR الكمي (qPCR) ، والكشف عن السيتوكينات وهرمون تستوستيرون عليها.

النتائج: أظهرت نتائجنا وجود كمية كبيرة من الطفيلي في الخصية، تليها زيادة السيتوكينات الموالية للالتهابات IL1-β، وTNF-α وIFN-γ، وهرمون تستوستيرون .وفي وقت لاحق، تم زيادة مقدار تواجد IL-4 في الخلية ولوحظ استمرار تواجد الطفيلي في الخصية باستخدام المنهج التجريبي.

الخلاصة: هاجرت اللَيشْمانَات خارج الخلية إلى البربخ لتشكل عاملا رئيسيا محتملا لاستمرار الطفيليات والانتقال التناسلي لعدوى *L. chagasi* في الهامستر.

Translated from English version into Arabic by Mahmoud Sami, through

TRANSLATORS
WITHOUT BORDERS

雄性仓鼠生殖系统感染恰加斯利什曼原虫的动力学特征

Amanda P N Quintal, Bruna C Borges, Paula C Brígido, Rebecca T Silva, Ana F Notário, Marlus A Santos, Maria A de Souza, Fernanda G O Nascimento, Antônio V Mundim, Guilherme M J Costa, André B Vasconcelos, Claudio V Silva

摘要

引言：在慢性感染期，利什曼病引起动物生殖系统改变和损伤，导致无精子症和睾丸萎缩。本研究旨在揭示雄性金色仓鼠（*Mesocricetus auratus*）生殖系统感染恰加斯利什曼原虫的动力学特征。

方法：动物腹腔接种恰加斯利什曼原虫无鞭毛体。在不同时间点处死动物，其生殖器官进行组织病理学检查、定量 PCR、细胞因子和睾酮检测分析。

结果：实验结果表明，睾丸虫荷高，促炎细胞因子 IL1-β、TNF-α、IFN-γ 和睾酮也随之增加。随后 IL-4 表达上调，并观察睾丸基部利什曼原虫的存留时间。

结论：细胞外恰加斯利什曼原虫无鞭毛体迁移到仓鼠附睾是寄生虫存活和性交传播的潜在重要因素。

Translated from English version into Chinese by Chen Jin, edited by Yang Pin, through

CHINESE CENTER FOR DISEASE CONTROL AND PREVENTION
NATIONAL INSTITUTE OF PARASITIC DISEASES

Révéler la cinétique de l'infection Leishmanias Infantum dans les parties génitales mâles chez les hamsters.

Amanda P N Quintal, Bruna C Borges, Paula C Brígido, Rebecca T Silva, Ana F Notário, Marlus A Santos, Maria A de Souza, Fernanda G O Nascimento, Antônio V Mundim, Guilherme M J Costa, André B Vasconcelos, Claudio V Silva

Résumé

Origine: La Leishmanias provoque des mutations et lésions dans le système génital qui donne lieu à l'azoospermie et l'atrophie testiculaire chez les animaux durant la phase chronique de l'infection .L'objectif de cette étude était de révéler la cinétique de l'infection Leishmanias Infantum du système génital mâle des hamsters dorés (*Mesocricetusauratus*).

Méthodes : Les animaux s'étaient fait inoculer de l'amastigote d'une manière péritonéale de Leishmanias Infantum. A des moments différents les animaux étaient euthanasiés et leurs organes génitaux traités via d'essai de dépistage histo-pathologique, QPCR, la cytokine et testostérone.

Résultats : Nos résultats ont montré qu'une charge élevée de parasites dans les testicules suivie par une hausse dans la Cytokine pro-inflammatoire IL1- β, TNF- α et IFN-y et la testostérone. En conséquence, l'expression IL-4 était régulée et la persistance parasitaire basale dans les testicules était remarquée utilisant l'approche expérimentale.

Conclusion: Les amastigotes extracellulaires migraient vers l'epididymis se posant en facteur potentiel de la persistance parasitaire et la transmission vénérienne de l'infection L-Infantum chez les hamsters.

Translated from English version into French by CHAKIB ROULA, through

TRANSLATORS
WITHOUT BORDERS

Выявление движения инфекции *Leishmania chagasi* в половой системе самцов хомяков

Amanda P N Quintal, Bruna C Borges, Paula C Brígido, Rebecca T Silva, Ana F Notário, Marlus A Santos, Maria A de Souza, Fernanda G O Nascimento, Antônio V Mundim, Guilherme M J Costa, André B Vasconcelos, Claudio V Silva

Аннотация

Базовая проблематика. Лейшманиоз вызывает изменения и повреждения половой системы, что приводит к азооспермии и атрофии яичек у животных в хронической стадии заболевания. Цель данного исследования — выявление движения инфекции *Leishmania chagasi* в половой системе самцов сирийских хомяков *(Mesocricetus Auratus).*

Методы. В различные моменты времени животных подвергали эвтаназии, а половые органы — обработке для проведения гистопатологического исследования, количественной ПЦР, исследований на определение уровня цитокинов и тестостерона.

Результаты. Наши результаты показали наличие высокой паразитарной нагрузки в семенниках с последующим увеличением уровня провоспалительных цитокинов ИЛ1-бета, ФНО-альфа и ИФН-гамма, а также тестостерона. Как следствие, произошло повышение экспрессии ИЛ-4, и с помощью экспериментального подхода выявлена персистенция основной инфекции в семенниках.
Вывод. Внеклеточные амастиготы, мигрировавшие в придатки яичка, выступают основным потенциальным фактором возникновения персистенции и передачи венерического инфекционного заболевания *L. chagasi* у хомяков.

Translated from English version into Russian by Anna Romanenko, through

Revelación de la cinética de la infección *Leishmania chagasi* en el aparato genital masculino de hámsters

Amanda P N Quintal, Bruna C Borges, Paula C Brígido, Rebecca T Silva, Ana F Notário, Marlus A Santos, Maria A de Souza, Fernanda G O Nascimento, Antônio V Mundim, Guilherme M J Costa, André B Vasconcelos, Claudio V Silva

Abstracto
Antecedentes: Leishmaniasis causa alteraciones y lesiones en el sistema genital, lo que conduce a la azoospermia y atrofia testicular en los animales durante la fase crónica de la infección. El objetivo de este estudio fue revelar la cinética de la infección *Leishmania chagasi* en el aparato genital de hámsters dorados machos (*Mesocricetus auratus*).
Métodos: los animales fueron inoculados por vía intraperitoneal con amastigotes de *L. chagasi*. En diferentes puntos de tiempo, los animales fueron sacrificados y los órganos genitales procesados para ensayos histopatológicos, de qPCR, citoquinas y de detección de testosterona.
Resultados: Nuestros resultados mostraron una alta carga parasitaria en los testículos, seguida de un aumento de citoquinas proinflamatorias IL1-beta, TNF-alfa e IFN-gamma y testosterona. Posteriormente, la expresión de IL-4 se reguló y se observó la persistencia del parásito basal en los testículos utilizando el enfoque experimental.
Conclusión: los amastigotes extracelulares emigraron al epidídimo, presentándose como un factor potencial importante de persistencia del parásito y de la transmisión venérea de la infección *L. chagasi* en hámsters.

Translated from English version into Spanish by Álvaro de Marcos Peirotén, through
